# Patient views of the good doctor in primary care: a qualitative study in six provinces in China

**DOI:** 10.1186/s41256-023-00309-y

**Published:** 2023-07-11

**Authors:** Wenhua Wang, Jinnan Zhang, Jiao Lu, Xiaolin Wei

**Affiliations:** 1 School of Public Policy and Administration, Xi’an Jiaotong University, No. 28 West Xianning Road, Xi’an, People’s Republic of China; 2 Dalla Lana School of Public Health, University of Toronto, Toronto, Canada

**Keywords:** Health policy, Primary care, General practitioners, Quality of care, Qualitative research

## Abstract

**Background:**

China has been striving to train primary care doctors capable of delivering high-quality service through general practitioner training programs and family doctor team reforms, but these initiatives have not adequately met patient needs and expectations. In order to guide further reform efforts to better meet patient expectations, this study generates a profile of the good doctor in primary care from the patient perspective.

**Methods:**

Semi-structured interviews were conducted in six provinces (Shandong, Zhejiang, Henan, Shaanxi, Shanxi, Heilongjiang) in China. A total of 58 interviewees completed the recorded interviews. Tape-based analysis was used to produce narrative summaries. Trained research assistants listened to the recordings of the interviews and summarized them by 30-s segments. Thematic analysis was performed on narrative summaries to identify thematic families.

**Results:**

Five domains and 18 attributes were generated from the analysis of the interview data. The domains of the good doctor in primary care from the patient perspective were: strong Clinical Competency (mentioned by 97% of participants) and Professionalism & Humanism (mentioned by 93% of participants) during service delivery, followed by Service Provision and Information Communication (mentioned by 74% and 62% of participants, respectively). Moreover, Chinese patients expect that primary care doctors have high educational attainment and a good personality (mentioned by 41% of participants).

**Conclusions:**

This five-domain profile of the good doctor in primary care constitutes a foundation for further primary care workforce capacity building. Further primary care reform efforts should reflect the patient views and expectations, especially in the family physician competency framework and primary care performance assessment system development. Meanwhile, frontline primary care organizations also need to create supportive environments to assist competent doctors practice in primary care, especially through facilitating the learning of primary care doctors and improving their well-being.

## Background

Strengthening the service capacity of doctors in Primary Health Care (PHC) has been proposed as an efficient approach to transform the current hospital-centric delivery system into PHC-based delivery system in China [1, 2]. During the last decade, China has implemented various policies aimed at training more qualified primary care doctors and reforming the primary care delivery models to ensure high-quality service provision [3].

To train a sufficient number of qualified primary care doctors, in 2011, the State Council issued a national guideline to develop a unique general practitioner (GP) training program, which included both the long-term “5 + 3” GP residency training model (five-year undergraduate medical education followed by three-year standardized GP residency training) and short-term clinical specialty transfer training model (1-year training for existing primary care providers who want to register as GPs) [3]. To address the pressing challenges of an ageing population and the growing burden of chronic diseases, in 2018, the State Council set a new national target of providing five GPs per 10,000 population by 2030 and released another national guideline to enhance the quality of GP training programs [4]. The main policy efforts focus on encouraging medical schools to build departments/schools of general practice, provide mandatory courses in general practice, build general practice teaching sites, and develop training standard. The residency training hospitals are required to increase the number of general practice resident positions and build independent departments of general practice to provide general practice services. The guidelines also encourage the development of continuing medical education, including digital training courses and tele-education models. These training programs have achieved remarkable results, leading to the tripling of qualified GPs from 0.15 million in 2013 to 0.41 million in 2020 [5].

To transform PHC towards high-quality service provision, China has, since 2016, implemented reforms to support the building of the family doctor team model, which typically integrates family doctors, registered nurses and public health physicians to provide basic medical care services, public health services, and individualized health management to local residents in a community [6]. Meanwhile, performance assessment policies were developed to link the payment of practitioners in the family doctor team to the following performance indicators: number of patients signing contracts with the team, service quantity and quality, outcomes of health management, patient satisfaction, and cost, etc. [6]. Following the achievements of the family doctor team reform, in 2022, another national policy was issued to promote high quality family doctor team building, especially in expanding the sources of family doctors, strengthening family doctors’ clinical competency training, enlarging the scope of service provision, and optimizing the service delivery approach [7].

Despite the improvement of the service capacity of primary care doctors based on these reform efforts, evidence shows that Chinese residents still prefer to bypass PHC services when they seek health care. For example, the number of outpatient visits in primary care has increased by 25.40% (from 3.61 billion in 2010 to 4.53 billion in 2019) [3, 8–10], but the proportion of outpatient visits to primary care in the whole system has decreased from 61.87% to 2010 to 53.17% in 2020 [11, 12]. There is national consensus that the service capacity of primary care doctors failing to meet patient expectations is the key reason. As China is moving towards building a People-Centered Integrated Care (PCIC) system based on strong PHC, as suggested by World Health Organization (WHO) and the World Bank [13, 14], generating knowledge on patients’ expectations, values and preferences regarding the “good doctor” in primary care is important to ensure that patients’ voices are heard and reflected in further reform efforts in China.

Previous studies mostly focus on examining the patient expectations of good PHC in a broad perspective and are dominantly in Europe and North America [15–18]. Only few studies have been conducted to explore the patient expectation of good doctors in primary care setting. For example, a study from the Netherland found that patients had high expectation of good GP in areas of sufficient consultation time, availability of appointments at short notice, and being given detailed information about their illness [19]. A UK study found that the patients placed higher priority on the technical quality of care and continuity of care with their primary care physicians [20]. A recent study in Israel reported that the most important attributes of a “good doctor” from the public perspective are knowledge and professionalism, credibility and honesty, humaneness, listening and patience [21]. However, there are differences between countries in the importance patients attach to aspects of healthcare [22]. Evidence suggests that health system investments, culture and the human development context have stronger influences on patients’ priorities for their health systems than individual level factors such as age, sex, education, health status, and utilization of health services [16, 23]. Therefore, it is necessary to generate knowledge based on China context. In this study, we undertook a qualitative study in six Chinese provinces to generate the profile of the good doctor in primary care in China.

## Methods

### Study design

A qualitative descriptive study using semi-structured interviews was conducted among primary care users from six provinces (one city in each province), including the eastern region (Jinan City in Shandong Province and Hangzhou City in Zhejiang Province); the central region (Luoyang City in Henan Province, Taiyuan City in Shanxi Province and Harbin City in Heilongjiang Province); and the western region (Xi’an City in Shaanxi Province) [24].

### Interview guide development

The interview guide was initially developed based on a literature review, followed by a pilot study among 15 primary care users in Xi’an in August 2020 and expert discussion to ensure appropriateness, relevance and linguistic quality. The interview guide included the following sections: general information on the participants; participants’ health and previous care seeking behaviors; and what constitutes “good doctors in primary care” from the participants’ point of view.

### Participant recruitment

Eligible participants were 18–80 years old, with no cognitive impairment and having had experiences of visiting doctors in local primary care settings during the past 12 months. Recruitment was facilitated by local government officials through purposive and snowball sampling. The “sampling to theme saturation” strategy, which involves a flexible approach dependent on ongoing data analysis and generating new conceptual ideas to test against the primary data, was used [25]. Initially, we planned to recruit between 8 and 12 participants from each city, which has been identified as a sufficient sample size to identify the themes that emerge as most common to the whole group [26]. The sampling continued until no new themes transpired in the interviews. Our final sample was 58 participants: 11 from Shandong, 11 from Zhejiang, 8 from Shaanxi, 8 from Henan, 8 from Shanxi and 12 from Heilongjiang.

### Data collection

After obtaining informed consent from participants, semi-structured interviews were conducted by trained research assistants from April to December 2021 using the tested open-ended qualitative interview guide. During the interview process, our interviewers first asked participants about their health care seeking experiences during the past year. Then, the interviewers encouraged the participants to speak in their own words about their views and expectations for the good doctor in primary care. Such an approach provided flexibility to explore the interviewee’s own values and meanings without imposing preconceived structures, assumptions and language [27]. The interviewers provided interviewees with an explanation of the relevant terms prior to the start of the interview. The interviews were conducted face-to-face, one-on-one and lasted between 30 and 60 min. With the consent of the interviewees, all semi-structured interviews were audio-recorded. Immediately following the interview, interviewer completed debriefing notes to capture contextual details of the interview, including non-verbal communication and the interview process [28]. Interview recordings were uploaded into NVivo12 software.

### Data analysis

Thematic analysis was conducted to analyse the interview data [29, 30]. Our analysis was predominantly descriptive, combining deductive and inductive approaches to allow original themes to emerge.

We used tape-based analysis which is more efficient but equally rigorous in comparison to more traditional transcription-based analysis. It allows the analyst to maintain a rigorous and consistent awareness of the coding structure, including continual visual referencing of the coding structure while listening to the interview [31, 32]. Trained research assistant listened to the recording of the interviews and summarized them for each 30-s segment. In addition, the research assistants noted themes from the analytic framework or made notes about the behaviour of the interviewee, such as hesitations, lack of comfort, refusal to answers or tangents. The narrative summary preserved the narrative flow of the interview, while showing relevant quotations or transcriptions and parallel analytic notations as well as facilitating the deidentification of data to protect participant confidentiality. This data analysis approach has been tested and used by other qualitative researchers [33].

Next, thematic analysis was performed. Codes were initially derived independently by two analysts. The analysts had several meetings to discuss coding and finalize the codebook. Once the codebook was established, one analyst continued to code the remaining narrative summaries, which were then verified by the second analyst [24, 34]. Any disagreements between the two analysts were discussed with the whole research team, to reach consensus.

The codes were initially assigned to the following domains: communication and interpersonal skills, professionalism & humanism, diagnostic acumen, practice scope, and personal characteristics, based upon existing frameworks and relevant studies regarding good doctors in primary care [35–40], while considering our previous study about patient perspectives on primary care in China [41]. The codes in each domain were then categorized into sub-domains (attributes) based on their meaning in the Chinese context. We also modified the structure and components of domains following the analysis process. Three group discussions within the research team were conducted to finalize the final profile of good doctors in primary care from the patients’ perspective.

## Results

### Participant characteristics

Table 1 summarizes the characteristics of the 58 participants who had visited community health centers in urban China within the past 12 months. Half of the participants were female and had a college education or above; 80% were under 60 years-old; 25% had one or more chronic diseases; and 71% were covered by urban employee basic medical insurance (UEBMI), for urban employed and retired, with the rest covered by urban and rural resident basic medical insurance (URRBMI), which covers the unemployed, students and children. We performed subgroup analysis of patients’ view of the good doctor by age, sex, medical insurance, chronic disease status (yes/no). The results did not vary across subpopulations.

**Table 1:** Characteristics of participants (*N* = 58)

Characteristic	No. (%)
Age, mean (SD), years	45 (14.3)
< 60	47 (81.0)
≧ 60	11 (19.0)
Sex	
Male	29 (50.0)
Female	29 (50.0)
Education level	
Primary school or below	4 (6.9)
Junior high school	10 (17.2)
Senior high school	14 (24.1)
University or college	20 (34.5)
Master’s degree or higher	10 (17.2)
Medical insurance	
Urban employee basic medical insurance	41 (70.7)
Urban resident basic medical insurance	17 (29.3)
Chronic diseases status*	
Yes	14 (24.1)
No	44 (75.9)

*Chronic diseases here only included diabetes and/or hypertension

### Five domains of the good doctor in primary care from the patient perspective

The following five domains including 18 attributes were generated from the analysis of the interview data: Clinical Competency (mentioned by 97% of participants), Professionalism & Humanism (mentioned by 93% of participants), Service Provision (mentioned by 74% of participants), Information Communication (mentioned by 62% of participants), and Personal Characteristics (mentioned by 41% of participants). Table 2 presents the five domains, their attributes, and poignant quotes.

From the patients’ perspective, the good primary care doctor with strong Clinical Competency should hold professional qualification in general practice with rich clinical experience and comprehensive medical knowledge, could make a quick and accurate diagnosis, and prescribe effective treatment to quickly relieve a patients’ symptoms. Meanwhile, the good doctor is also expected to have strong competency in Professionalism & Humanism, which means she or he should treat patients with good service attitude while maintaining medical professionalism. At the same time, he or she should demonstrate respect, patience, and empathy toward their patients and have a comprehensive knowledge of their patients from a social determinant of health perspective. Besides Clinical Competency and Professionalism & Humanism, the good primary care doctor is also expected to have strong Information Communication competency, which means that he or she should gather sufficient information on the patients and their conditions, through proactively asking questions and patiently listening to their patients, and give clear explanations about the conditions, treatment and prescriptions. The Service Provision domain suggests that the good doctor with these strong competencies is expected to provide services including treatment of common diseases, public health services, especially health management and follow-up services, through an efficient approach involving the same doctor providing services each time, appropriate referrals, out-of-hour services, and online consultation. Finally, the good primary care doctor is a doctor with a good personality who is middle-aged and has at least a bachelor’s degree.

## Discussion

As China is moving towards building a PCIC model of care based on strong PHC, further reform efforts are needed to train competent primary care doctors and improve their performance. These reform efforts should reflect patients’ views and expectations of good doctors in primary care. Based on in-depth semi-structured interviews with primary care users in six provinces, this study has generated a clear profile of the good doctor in primary care from the patient’s perspective in China. Chinese patients expect their primary care doctors to have strong Clinical Competency, Professionalism & Humanism, followed by Service Provision and Information Communication. In addition, Chinese patients also expect their primary care doctors to have comprehensive medical knowledge, high educational attainment and good personality.

Clinical Competency is the most valued domain of the good primary care doctor by Chinese patients. This result is consistent with studies in other countries [18] and the recent studies in China [42, 43]. However, previous studies did not provide the operational definition of clinical competency in the Chinese context. Based on the data from six provinces, this study isolated five attributes of clinical competency, presenting a clear picture of what clinical competency entails from the perspective of patients in China. A good doctor with strong clinical competency should be a general practitioner with rich clinical experiences, comprehensive medical knowledge and high clinical skills, who could make quick and accurate diagnosis, and provide quick relief of the patients’ symptom. These attributes have been positively linked in other studies with high quality care [44–49], and have also been shown to be strong predictors of patients’ choice of primary care providers [50, 51].

The Professionalism & Humanism domain of the good doctor in primary care, which is valued by most participants, includes six attributes: good service attitude, respect, patience, empathy, professionalism, and knowledge of patient. This six-attribute structure gives the operational definition of Professionalism & Humanism domain in Chinese primary care. This domain is also highly valued by the patient population in other countries. For example, a previous systematic review summarizing patient priorities for primary care studies published before 2000s reported that humanism was the most important aspect from patient views, which was ranked highest in 86% of the studies that included this aspect [18]. A recent literature review on how patients want their doctor to communicate also revealed that most patients expected their doctors to be friendly, respectful, interested, non-judgmental and sensitive and to treat patients as a person and as a partner [39]. Recognizing the importance of this domain, humanities medical courses have been offered in medical school, and this domain has been included in recent studies on the competency of Chinese general practitioners [52].

Our study participants suggested that the Information Communication domain of the good doctor in primary care should involve two processes: gathering information and providing information. During the information gathering process, patients expected that their doctor inquire about their illness in detail and listen to them for a sufficient length of time. Before making a treatment decision, patients value a detailed explanation of their condition and treatment plan. This is consistent with evidence from Lithuania, Malaysia, Sweden, Hungary, and Poland [53–57]. Also, this corresponds to the international call for patients’ active participation in decision-making, which requires doctors to be good at identifying patient needs and patients to be brave enough to clearly express their wishes and seek consensus on treatment together [58]. Good communication, such as better listening skills, could not only influence patient decision to see the doctor, but also further enhance patient adherence to treatment regimens and result in better health outcomes. There is extensive evidence that good communication is associated with improved clinical outcomes, reduced medical errors, and facilitated patient self-management and preventive behaviors [59–61].

**Table 2:** Five domains, 18 attributes and their exemplar quotes generated from qualitative interviews among 58 participants

Domains	Attributes	Exemplar quotes
Clinical competency (56)	Experience (45)	“Experienced physicians know more about diseases, and they know what it is once they grasp the patient’s symptoms.” (LY7) “A good doctor should work for more than five years.” (LY6) “The professional capacity is the most important thing for doctors, and the expertise of doctors in community health centers is still much worse than that of hospital doctors.” (JN5) “(Firmly)
	Qualification (23)	“Doctors in the community are certainly not allowed to work without certification. But now the state regulation is stricter, allowing doctors with medical qualifications to work in the community.” (JN2) “Some community doctors came from hospitals and were professionally trained. This gives the impression that the doctor is very professional.” (LY6)
	Knowledge (22)	“Community doctors should be clinically well rounded, with knowledge of various specialties such as internal medicine, surgery and gynaecology.” (TY4) “Community health center physicians should be proactive in learning new medical knowledge.” (HZ5)
	Effectiveness (16)	“I expect a positive change in my condition as a result of the doctor’s treatment.” (LY1)
	Diagnose acumen (14)	“I don’t require doctor to prescribe medication that works instantly. But he should at least be able to prescribe the right drugs for the symptoms.” (LY2) “(Firmly) “A good doctor can identify the disease based on my symptoms in a short period of time through effective communication.” (XA7)
Professionalism & humanism (54)	Service attitude (38)	“Doctors should treat patients kindly during consultations and not make them feel anxious.” (HRB12) “The attitude of community health center doctors could learn from doctors in private medical institutions, that is, treating patients more warmly.” (JN5)
	Professionalism (19)	“Doctors should take simple tasks such as giving vaccinations and measuring height and weight seriously as well.” (TY5)
	Empathy (13)	“Doctors at community health centers in cities are very nice to patients. (Sigh) But the attitude of doctors at township health centers in rural areas is relatively not very good. Good doctors are able to put themselves in the patient’s shoes and comfort and encourage them.” (JN2)
	Respect (13)	“I hope the doctor could consider my views when prescribing medication. (Smile bitterly)” (XA1)
	Knowledge of patient (11)	“Doctors should consider the patient’s personal situation and ask if they are allergic to certain drugs. They should prescribe drugs according to symptoms after asking about the patient’s condition.” (TY8)
	Patience (10)	“A good doctor should communicate patiently with patients.” (LY6) “Since the work of doctors in community health centers involves frequent contact with children, such as vaccinations. Doctors should show patience with children.” (TY5)
Service provision (43)	Practice scope (33)	“Family physicians can tell me how to prevent the disease by saying something like exercise more and avoid eating high-sugar fried foods. (Pause) Many diabetics in the community don’t know how to take care of themselves.” (XA3) “They should be able to handle various common diseases.” (HZ4) “It is important to follow up the patient. My doctor should call me a few days after I leave the community health center to ask if I’m getting better.” (HRB8)
	Practice approach (19)	“If the community doctor can’t handle it, he / she should inform the patient, suggest the patient to refer to the hospital, or recommend the doctor in hospitals to the patient.” (TY2) “I prefer to consult the same doctor who has diagnosed me before because he is familiar with my condition.” (HZ9) “Community health centers should build a community patient WeChat group where doctors are able to provide online guidance for community residents.” (TY6)
Information communication (36)	Information gathering (28)	“The doctor should ask the patient in detail about his or her symptoms.” (HRB4) “The doctor should listen to my description of the symptoms.” (HZ10)
	Information provision (20)	“I hope the doctor can explain to me how my condition may progress in the future.” (JN6) “The doctor should explain to me what each medication prescribed to me does.” (LY6)
Personal characteristics (24)	Age (19)	“There should be more middle-aged and elderly doctors in the community, because they are more senior. Young doctors are less experienced.” (XA3)
	Education (15)	“I expect doctors in community health centers to have at least bachelor’s degrees, preferably master’s degrees.” (HZ6)
	Personality (5)	“(Smile) It is best for community doctors to have a milder disposition. The doctors in my community are good-tempered.” (JN3)

The brackets identify the number of participants expecting this domain/attribute, and the city of the exemplar quote: LY is Luoyang; HZ is Hangzhou; XA is Xi’an; JN is Jinan; TY is Taiyuan; HRB is Harbin

Transforming the hospital-centric system into primary care-based delivery system is a key component of further national reform efforts. From the patients’ perspective, the good primary care doctor should provide services of common disease treatment and public health, especially health management and follow-up through a continuous and coordinated approach, which involves the same doctor providing services each time as well as appropriate referral services, out-of-hour services, and online consultation. As China is moving towards building PCIC system, the family doctor team, providing integrated primary care and public health services based on local community residents’ health needs, is one of the key reform efforts to better meet patients’ needs and expectations, especially in the service provision domain. In fact, the Service Provision that patients expect in our study is also the service content provided by the Chinese family doctor team. The recent national policy also proposes to expand the scope of services by primary care doctors and provide services through in-person and online approaches based on different digital tools. Further policy evaluation is needed to examine if these policies could better meet patients’ expectations.

Personal Characteristics, such as high educational attainment and age-related maturity, were also highlighted by patients. Age-related expectations, which link age, experience and skill levels, are deeply rooted in the Chinese culture, even though age and performance are not necessarily positively correlated [62, 63]. According to Chinese physician recruitment rules, doctors with lower education and experience are more likely to work in primary care institutions than hospitals. The majority of these physicians therefore hold medical degree at undergraduate level or below, which means lower Clinical Competency from the patient perspective. This may be one of the reasons why patients prefer the hospital system when they seek care [3, 64].

Our study findings have several implications for practice and policy. China has a strong political commitment to build a PCIC system based on high-performing PHC system. Training, recruiting and retaining qualified doctors in primary care is a foundational approach. The five domains and their specific attributes of the good primary care doctor could help guide further initiatives to strengthen family medicine education and practice. First, a national family physician competency framework should be developed to guide medical school training and residency training. The five-year medical school training program in China is currently mainly based on medicinal theories and clinical medicine, as well as basic knowledge of preventive medicine. The main target of the three-year residency training program is to improve clinical and public health practice competency, and training evaluation focuses on numbers of diseases, numbers of cases, and clinical and public health practice competencies. Information Communication competency as well as Professionalism & Humanism are not emphasized in these programs. For continuing medical education, the most frequent training topics are basic clinical theory knowledge and clinical practice skills [65], although doctors in primary care in China have a great need for communication skills training [65]. This framework could define the abilities needed from family physicians across the educational continuum of undergraduate, postgraduate, and continuing professional development, especially in Clinical Competency, Professionalism & Humanism and Information Communication, which are prioritized by patients. Second, Chinese patients expect the good primary care doctor to have comprehensive medical knowledge and constantly learn to update their knowledge, which points out the importance of building a learning primary care system. Local primary care organizations should be supported and encouraged to build learning organizations through need-based continuing professional development programs. Third, daily management practices of primary care organizations should also be further reformed to support wellbeing of primary care doctors. For primary care practices, low job satisfaction and high occupational burnout are widespread among primary care doctors in China [3]. One of the reasons is that China’s primary care doctors have to undertake both medical and public health work, with heavy workloads making it difficult to devote sufficient time for each patient to communicate and show more humanism during clinical encounters [66]. Appropriate digital health technologies as well as a supportive organizational culture could facilitate this reform process.

Our study results need to be interpreted in light of its limitations. There is a high demand for health care among the rural population. Their expectations were not explored in this study. Future studies could investigate the preferences of rural populations for doctors in primary care and compare them with those of urban populations. Second, despite rigorous methods to maintain trustworthiness, translation of original statements into English for publication purposes may have resulted in some linguistic inconsistencies. Finally, participants were asked to recall their past experiences, which may have impacted the accuracy of their accounts.

## Conclusions

Guided by Chinese patients’ views, this study has generated a profile of the good doctor in primary care that includes five domains: Clinical Competency, Professionalism & Humanism, Service Provision, Information Communication, and Personal Characteristics. Further primary care reform efforts should reflect these patients’ expectations. Ongoing family medicine education reform should consider building a national family physician competency framework to guide medical school training and family medicine residency training, especially in areas of Clinical Competency, Professionalism & Humanism, and Information Communication. Further management practice reform within primary care organizations is also needed to provide a supportive organizational context for competent doctors to practice in primary care and continue their learning.

## Data Availability

Data underlying this article cannot be shared publicly to protect the privacy of individuals who participated in the study. Aggregate data will be shared on reasonable request to the corresponding author.

## Abbreviations

PHC: Primary Health Care
GP: General Practitioner
PCIC: People-Centered Integrated Care
WHO: World Health Organization
UEBMI: Urban Employee Basic Medical Insurance
URRBMI: Urban and Rural Resident Basic Medical Insurance
